# The History of Bancroftian Lymphatic Filariasis in Australasia and Oceania: Is There a Threat of Re-Occurrence in Mainland Australia?

**DOI:** 10.3390/tropicalmed3020058

**Published:** 2018-06-04

**Authors:** Catherine A. Gordon, Malcolm K. Jones, Donald P. McManus

**Affiliations:** 1 Molecular Parasitology Laboratory, QIMR Berghofer Medical Research Institute, Brisbane, QLD 4006, Australia; Don.McManus@qimrberghofer.edu.au; 2 School of Veterinary Science, University of Queensland, Brisbane, QLD 4072, Australia; m.jones@uq.edu.au

**Keywords:** *Wuchereria bancrofti*, lymphatic filariasis, elephantiasis

## Abstract

Lymphatic filariasis (LF) infects an estimated 120 million people worldwide, with a further 856 million considered at risk of infection and requiring preventative chemotherapy. The majority of LF infections are caused by *Wuchereria bancrofti*, named in honour of the Australian physician Joseph Bancroft, with the remainder due to *Brugia malayi* and *B. timori*. Infection with LF through the bite of an infected mosquito, can lead to the development of the condition known as elephantiasis, where swelling due to oedema leads to loss of function in the affected area and thickening of the skin, ‘like an elephant’. LF has previously been endemic in Australia, although currently, no autochthonous cases occur there. Human immigration to Australia from LF-endemic countries, including those close to Australia, and the presence of susceptible mosquitoes that can act as suitable vectors, heighten the possibility of the reintroduction of LF into this country. In this review, we examine the history of LF in Australia and Oceania and weigh up the potential risk of its re-occurrence on mainland Australia.

## 1. Introduction

Lymphatic filariasis (LF), also known as Bancroftian filariasis or elephantiasis—due to swelling often in the lower limbs and genitals, upper limbs, and other areas of the body—is part of Australian history, with father and son, Dr. Joseph and Dr. Thomas Bancroft, two pre-eminent physicians and parasitologists, integral to the elucidation of the life-cycle of the human parasitic roundworm causing the disease. The causative agent of Bancroftian filariasis is a spirurid nematode *Wuchereria bancrofti*, named in honour of Joseph Bancroft and of Dr. Otto Wucherer, who was based in Brazil [1]. 

**Figure 1:**
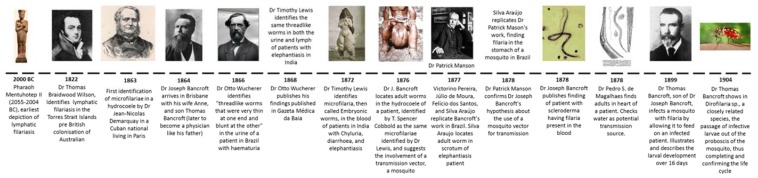
Timeline of Bancroftian filariasis showing the earliest known record in the form of a statue of Pharaoh Mentuhotep II (2055–2004) and through to the elucidation of the lifecycle finalised in 1904 by Dr. Thomas Bancroft.

The earliest evidence of LF comes from Egypt, with a statue of Pharaoh Mentuhotep II (2055–2004 BC) depicting swollen limbs, which are characteristic of the disease (Figure 1) [2], while artefacts from around 500 AD in West Africa also display scrotal swelling. The earliest written record of elephantiasis comes from the Ebers Papyrus (1550 BC) in Egypt [3]. The Greek medical writer, Celsus (30 BC–50 AD), also wrote about elephantiasis [3], the term as used then referring to both LF and leprosy [2], which can also present as elephantiasis due to the thickening of skin ‘like an elephant’. In later times, ancient Greek and Roman writers began referring to leprosy as ‘elephantiasis graecorum’ and LF as ‘elephantiasis arabum’ [2]. Similar descriptions of LF were provided by Chinese, Indian, Persian, and Arabian physicians from this time (500–600 AD). A Dutch merchant, Jan Huyghen van Linschoten, wrote of individuals in the Indian state of Goa (1588–1592) having ‘one of their legs and one foot from the knee downwards as thick as an elephant’s leg’, almost certainly describing LF [2]. 

Currently, there are 856 million people in 52 countries worldwide that are at risk of infection with the three species of nematodes which cause LF, including *W. bancrofti*, which accounts for 90% of LF cases [4], and *Brugia malayi* and *B. timori*, which are responsible for the remainder [5]. There are a number of other filarial nematodes, including zoonotic species, which can also cause infections in humans but do not present as LF [6]. In this review, we will be concentrating primarily on *W. bancrofti* only due to our focus on Oceania and the Pacific, where *Brugia* species are less common. *Brugia* species will only be referred to in areas where they are co-endemic with *W. bancrofti*.

**Figure 2:**
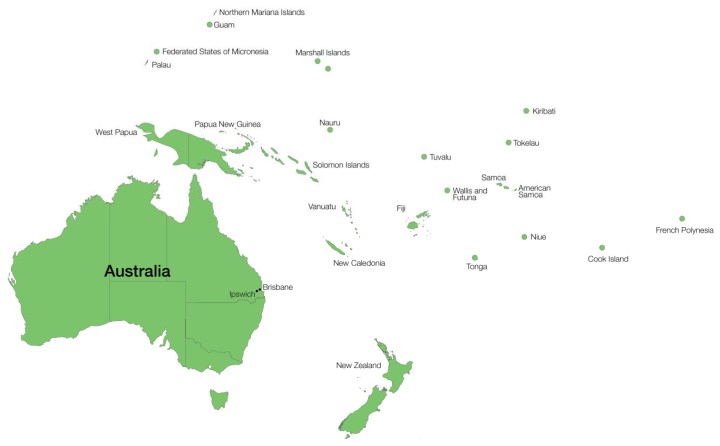
Location of the Islands present in Oceania and the Pacific referred to in this review.

In Australia, LF has been recorded historically along the eastern coast, extending from the Northern Rivers area of New South Wales to Far North Queensland and the islands of the Torres Strait (Figure 2). A focus was recorded for Brisbane, the capital city of the state of Queensland, where Bancroft practiced medicine [7]. Autochthonous cases have not been described since 1956 [8] in Australia, but returned travelers and service personnel, as well as immigrants and refugees, are a potential source of LF [6,9]. Our closest Pacific neighbours, Papua New Guinea (PNG) and Indonesia, remain endemic and could be the source of potential new cases and new invasive mosquito species capable of transmitting the disease. 

There are an estimated 2.7 million cases of LF in Oceania, accounting for 2% of the global disease burden due to this disease, although this figure comes from only one report of national data in PNG [10]. However, this may be an overestimate as prevalence varies greatly by village and province [11]. Utilising the Global Program for Elimination of Lymphatic Filariasis (GPELF) criteria, 4.81 million individuals in PNG live in endemic districts [11]. In the 2016 progress report, the number of people requiring preventative chemotherapy was 14.7 million in 11 countries of the Western Pacific [12]. While autochthonous cases of LF no longer occur in Australia, research has continued at Australian research institutes and Universities, notably at James Cook University, where the late Professor Rick Speare, whom this special issue is commemorating, was heavily involved in filariasis studies. Primarily this work revolved around diagnostics and the status of LF in PNG, which will be considered further later. 

The GPELF elimination strategy has two components. The first is to achieve transmission interruption, whereby the infection is not spread to new individuals, and the second is to control morbidity by alleviating the suffering of those who are or have previously been infected. When we talk of elimination in this paper, we refer to this definition by the WHO GPELF program [13].

## 2. Lifecycle 

**Figure 3:**
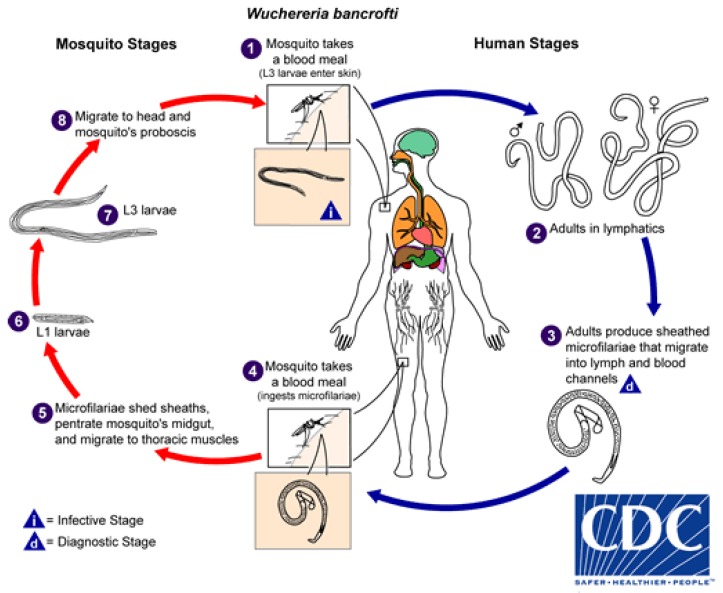
Life cycle of *Wuchereria bancrofti*. Image courtesy of the Centers for Disease Control and Prevention [14].

Although explored more fully below, the life-cycle is presented here. *Wuchereria bancrofti*, the causative agent of Bancroftian LF worldwide, requires two hosts to complete its life cycle; the human host, in which sexual reproduction occurs and the mosquito host, where maturation of L1 larvae to the infective L3 stage occurs (Figure 3) [14]. 

Adult worms sexually reproduce in the lymphatic system of an infected human. The adult females produce microfilariae (mf) which migrate into the blood. The mf are then taken up by a mosquito as it takes a blood meal and the mf mature into L1 larvae through to the infective L3s, which are deposited on to the skin of the human host during mosquito feeding [14] (Figure 3). It takes roughly 12 months for adult worms to mature and begin producing mf.

## 3. Disease

The pathology which manifests in the human host is varied, and a small proportion of long-term chronic cases can develop to the state that is referred to as elephantiasis, in reference to the swelling and thickening of the skin that can occur as a result of infection. Lymphedema, lymphangitis, lymphadenitis, funiculitis, cellulitis, chyluria, and hydrocele, swelling of the scrotum, and recurrent episodes of acute dermato-lymphangio-adenitis (ADLA) can also occur. There is a range of rare inflammatory and obstructive manifestations, including arthritis and myositis, pericardial effusion, and pericarditis. Development to overt clinical disease can take many years despite the presence of large numbers of mf in the blood, while individuals showing overt disease may have very low mf. This may be due to the adult worms no longer reproducing, which occurs after five years, single sex infections, adult worms still maturing, or as a result of the host immune response clearing infection [15]. 

The most common acute clinical manifestation is filarial fever, and the most common chronic manifestation is scrotal hydrocele [2]. Filarial fevers can occur without any other symptoms of LF infection and are due to toxins and allergens released by the parasite and secondary bacterial infection. Filarial fever begins with rigor and tremor, persisting for 1–3 h, and may be associated with congestion. Vomiting during an attack is symptomatic of retroperitoneal lymphatic involvement [2]. Scrotal hydrocele is a swelling in the scrotum that occurs when fluid collects around the testes and may or may not be preceded by other symptoms of LF infection, such as funiculitis [2]. In some cases, hydrocele will disappear following LF treatment or it may grow and progress. Treatment with doxycycline may cause a reduction in the size of smaller hydroceles, which is not seen with drugs used in mass drug administration (MDA). In general, large hydroceles require surgery [2].

In the years following infection while external symptoms may be low, considerable internal changes can be occurring, which may be responsible for the later overt physical disease symptoms, should they occur at all. The presence of adult worms is associated with the dilation of lymph vessels, lymphangiectasia [16]. This effect can be seen in areas of the lymph system where the adult worms are not physically present, which indicates that secretory/excretory parasite products may be present and have a more systemic effect [16]. The Gram-negative endosymbiont bacterium *Wolbachia* may also play a role in disease and contribute to inflammation following the death of adult worms and the subsequent release of the bacteria into the blood and lymph [16]. Patients who go on to develop lymphedema may also have a genetic predisposition, with lymphedema occurring in family groups [17]. However, clustering in family groups may be due to environmental conditions predisposing towards LF infection. Individuals with lymphedema in a Haitian study had higher levels of filarial antigens compared to those who did not have lymphedema [17], although this could be due to other factors, including disease stage.

ADLA can occur due to the death of adult worms, which can result in nodules or lesions in lymphatic vessels, or due to secondary bacterial infection, primarily by streptococci, which cause fissuring and loss of skin integrity [16,18,19]. During ADLA attacks, bacteria can be found in the blood and lymph [17]. Secondary infections tend to become more common as lymphedema progresses; however, they can be mediated by limb hygiene measures, which have been shown to help prevent ADLA attacks [20]. Fungal infections may help precipitate ADLA attacks by providing entry points for bacteria [18]. ADLA is an important step in the progression to lymphedema and elephantiasis, with an initial ADLA attack preceding and precipitating lymphedema, and subsequent ADLA attacks causing worsening lymphedema [18]. Additional ADLA symptoms include fever, chills, headache, vomiting, pain in the affected area, and vomiting. Severe cases can lead to toxaemia, altered senses, and urinary incontinence.

Tropical pulmonary eosinophilia (TPE) syndrome is also associated with filarial infection, although it is quite rare, with less than 1% of infections thought to lead to TPE [21]. The main symptoms include cough, shortness of breath, and wheezing, which may lead to long-term respiratory defects due to scarring of the lungs, resulting in restrictive and obstructive pulmonary abnormalities [21]. TPE occurs as a result of hypersensitivity to the filarial antigens of *W. bancrofti* and *Brugia* species, although it may also be due, in some cases, to zoonotic filarial infections as well [21]. TPE may occur in infected individuals due to sensitivity to the filarial antigen, which may trigger asthma [19]. 

## 4. Diagnosis

The two most common forms of diagnostic procedures for *W. bancrofti* detection are peripheral blood smears to identify mf, and antigen detection assays that test for parasite antigens in peripheral blood; there are no antigen detection tests for *Brugia* spp. [22]. Other diagnostic tests include antibody-based assays, DNA detection by polymerase chain reaction (PCR)-based assays, and mf membrane filtration. The most common antigen detection test currently employed in control programs is the filariasis immunochromatographic test (ICT) which was developed for field application. Both the ICT and mf detection have been employed in the 1998-launched WHO Pacific Programme to Eliminate LF (PacELF), the Pacific arm of the Global Programme, which aims to eliminate LF as a public health problem by 2020. The ICT has been replaced in the program with the filariasis test strip (FTS) diagnostic. One of the complications with mf detection is the nocturnality of *W. bancrofti*, necessitating that blood is drawn at night in order to provide the best opportunity to pick up the larvae [23]. In the Pacific, however, the strain of *W. bancrofti* that occurs does not demonstrate nocturnality [8]. The mf have difficulty passing through peripheral capillaries and are only seen in the peripheral blood at night, when they have the greatest activity [23]. During the day, when they are less active, they are unable to pass through the capillaries due to the reduction in their activity levels [23]. The advantage of immunodiagnostic procedures, both for antigen and antibody detection, is that blood can be drawn at any time. Circulating cell-free DNA (cfDNA) would also be detectable at any time, although night blood will contain larvae, thereby providing more DNA for detection, resulting in increased sensitivity [24,25].

### 4.1. ICT Antigen Test

The ICT antigen test, previously sold as the BinaxNOW Filariasis card test, was developed in 1997 by an Australian company (ICT diagnostics; New South Wales) and is a rapid test that detects soluble antigens of *W. bancrofti* circulating in the blood [26,27]. The test can be performed in the field and a result is obtained in 5–15 min; no preparation of blood or serum is required [26]. The ICT is in the form of a card with two pads, one of which is for the addition of serum (50 µL) and the other for a kit reagent. The card is then closed and the result viewed through a ‘window’ in the card. A positive result shows as a line underneath a control line [26]. Initial studies suggested that the test was 100% specific and highly sensitive [26], but there is evidence of cross-reactivity of the test with antigens from the related filarial species *Loa loa* in Africa [28,29]. In areas where *W. bancrofti* is the only filarial nematode present, this does not pose a problem, but when multiple filarial species are present, this will impact on the prevalence and distribution results. This test has been phased out and is no longer available. It has been replaced by the filariasis test strip (FTS) [30].

### 4.2. Filariasis Test Strip (FTS)

The ICT was used widely in the beginning of the GPELF but has been replaced by the FTS. Like the ICT, the FTS is a point-of-care test that detects *W. bancrofti* antigens. The test comes as a kit containing a test strip, work tray, and micropipette [31]. The micropipette is used to collect 75 µL of blood from a fingerprick. The blood is applied to the sample pad on the strip and left for 10 min, before the result is ready to be read. To be valid, the control line must be present. A valid positive result is two lines appearing, one control and one test. A valid negative result will only show the control line. The FTS was developed by Alere, the same company that manufactured the ICT, and was funded by a Bill and Melinda Gates Foundation grant [32]. In comparisons of the two tests, there was 99% agreement, while the FTS was more sensitive at lower antigen levels [31,32]. In addition to higher sensitivity at low antigen levels, the FTS is cheaper and has a longer shelf life [31].

### 4.3. Blood Smears (mf Detection)

Blood smears detect mf in the blood of an infected patient. As mentioned above, mf may not be present in patients with overt disease for a number of reasons, including single sex infections, the death of adult worms, adult worms no longer reproducing, or immature worms [15]. There are two types of blood smear that can be performed; a thick smear and a thin smear, and usually multiple slides are prepared to enhance the chances of finding the mf. Slides are stained with Giemsa to facilitate detection. Three-line thick smears are recommended for LF detection and involve three lines of blood of approximately 20 µL each placed horizontally next to each other on the slide and stained with Giemsa [8,15]. Blood can also be filtered through a membrane (3 µm pore size) and the membrane examined directly, although this is discouraged due to potential infection from aerosolised blood as it is forced through the membrane [15]. Concentration methods such as the Kontt’s or modified Knott’s technique are safer and allow for screening larger volumes of blood [15,19].

### 4.4. Other Diagnostic Methods

While the ICT and blood smears are the most common procedures used, there are a number of other available diagnostic tests including molecular- and serological-based assays. Serological tests include ELISA and CELISA (Cellabs Pty Ltd., Manly, Australia), an assay developed in Australia at James Cook University [33], which have been validated using dried blood spots (DBS) as well as the usual serum samples. The CELISA is also sold by TropBio (James Cook University, Townsville, Australia) as the Og4C3 antigen test which is considered a gold standard test for mf detection [34]. Og34C filter paper combined with ELISA has been shown to be more sensitive than ICT in a trial in PNG [35]. DBS have also been utilised for the DNA detection of LF by PCR [36].

*Wolbachia* levels in infected humans can be used to monitor the effect of drugs against adult worms, with levels reducing after the adults are killed [37,38]. 

## 5. Prevention and Treatment 

One of the best methods of thwarting the spread of a vector-borne disease involves targeting the vector, so as to prevent the mosquito from biting but also as a general mosquito population control measure. Methods used to prevent biting include the application of a personal repellent and the use of treated or untreated bed nets [39,40]. Mosquitoes generally need to bite a person upwards of several thousand times before LF infection occurs [15,41]; this is unlike malaria and other blood-borne pathogens, where infection can occur after far fewer bites. Studies in areas before and after the introduction of bednets have shown a reduction in mf prevalence in surveyed mosquitoes, indicating a decrease in LF transmission by mosquitoes through the introduction of bednets [40]. 

Treatment for LF is currently under the remit of mass drug administration (MDA). The WHO states that MDA should be undertaken in endemic areas and include everybody over two years old, except for pregnant women and those who are unwell [42]. To eliminate LF, multiple rounds of MDA should be undertaken annually, for a period of at least five years. This time frame was chosen as this is the expected reproductive lifespan of adult filarial worms in infected humans [42]. As chemotherapy has limited effects on the adult worms, it is possible to retain adults post-treatment that become less reproductively active until five to six years of age, at which stage they are considered reproductively inactive [43,44,45]. It is thus possible to be infected with adult worms that are no longer producing mf, but will be positive by serology. Previously, the main drug regimens in MDA was a combination of diethylcarbamazine citrate (DEC) with albendazole, albendazole alone, or a combination of ivermectin with albendazole [13,37]. In 2017, this was updated to a recommended three-drug treatment known as IDA, a combination of ivermectin, diethylcarbamazine citrate, and albendazole [46]. It is thought that the use of IDA drug combination will accelerate the global elimination of LF. More recent studies have indicated doxycycline and rifampicin, antibiotics that target *Wolbachia* spp., as a potential treatment, with high adult worm killing in patients given these drugs [43]. However, as doxycycline is an anti-malaria drug, it is unlikely to be approved for use in malaria-endemic areas due to the high potential for drug resistance exhibited by *Plasmodium* spp. parasites. MDA treatment has also been shown to reverse sub-clinical pathology in children [16].

ADLA can be treated with antibiotics to clear bacterial infection, bed rest, elevating the affected limb, and paracetamol [18,20]. Further attacks can be prevented by practicing hygiene and washing of the affected limb with soap daily, and treating any skin injuries quickly and appropriately with antibiotic ointments. Keeping the skin dry and using antifungal creams can prevent the development of fungal infections, which can provide entry of bacteria. The extent of lymphedema will determine how effective this treatment can be in preventing ADLA attacks due to the difficulty in washing deep skin folds, particularly with thickened, pitting skin which occurs in higher grades of lymphedema [18]. 

Lymphodema can be treated by bandaging or stocking, limb elevation at night, exercise of the affected limb, massage, intermittent pneumatic compression of the affected limb, heat therapy, and surgery [18,47]. 

## 6. History of LF in Australia

The work of unravelling the lifecycle of LF relied on observations of a number of physicians and parasitologists over many years (Figure 1). In 1863, the surgeon Jean-Nicolas Demarquay observed mf from a hydrocele of an infected Cuban national living in Paris [48]. This observation was followed in 1866 by the identification of threadlike worms in the urine of a patient by Dr. Otto Wucherer, working in the Brazilian state of Bahia [1,48,49]. Wucherer published his findings in the *Gazeta Médica da Bahia*, a journal not widely distributed at the time, and his important discoveries were not read by many others in the field, particularly in Europe. When Dr. Joseph Bancroft, working as physician in what is now the central business district of Brisbane, Australia, sent mf to Dr. T.S. Cobbold, working in the UK, via Dr. William Roberts, in 1874, who subsequently published his findings, calling the parasite *Filaria bancrofti*, it was without knowledge of the prior discovery by Dr. Wucherer (Figure 1) [50]. 

There was a divide between the Brazilian researchers and those in Europe, with the former taking aspects of European medicine deemed important for Brazil, but who were also keen to identify and deal with distinctive problems of health in tropical areas. There was also a divide between the older and younger generations within Brazil in how to deal with the many pressing health issues. This led to the founding of the Tropicalistas, a group initially made up of 14 physicians practicing in Brazil, and later known as the Bahian Tropical School, although no formal teaching occurred [51]. The group was dedicated to practicing and discussing modern medicine and its application to the tropical diseases that affected the poor in Brazil. Among the initial members were Otto Wucherer, John Paterson, and Jose Francisco da Silva Lima, who were at the forefront of the movement, and who helped propel a reformation of medical knowledge in Brazil, and indeed tropical diseases in general [49,51]. The *Gazeta Medica da Bahia* was published by the Tropicalistas to disseminate information and research findings [51], but as an essentially local publication, it had limited visibility outside of Brazil. 

### 6.1. Natural History in Australia

*Wuchereria bancrofti* (variously called *Filaria bancrofti* by T. S. Cobbold in 1876, and *Filaria sanguinis hominis* by P. Manson in 1878; *W. bancrofti* was not formalised until 1921) was initially found in Australia in the Torres Strait by T. B. Wilson in 1822 [7]. There are no known records, either written, in art, or in oral traditions of Australian Aboriginals (present in Australia for >40,000 years), for elephantiasis occurring on mainland Australia prior to colonisation by the British. It is possible that it was present at least in the Torres Strait due to the closeness of these islands to PNG and the movement of people between them. The first report of LF in the Torres Strait was made in 1822 [7]. A review of human filariasis in Australia published in 1986 suggested that LF in this country may have originated from PNG and was brought across to the Torres Strait by the movement of people between these islands. Further introduction of LF was facilitated by the immigration of individuals from LF-endemic areas, namely India, China, and the Pacific Islands [7,52]. British expatriates living in India invariably brought their Indian servants with them when emigrating to Australia, while Chinese immigrants began arriving in greater numbers from 1851 to the early 1900s with the advent of the Australian gold rush. Pacific Islanders were recruited as indentured labourers from 1863 to work on farms in tropical areas. From 1884 to 1901, these workers were restricted to working in tropical and sub-tropical localities unless they had been in the colony for more than five years [7]. Thus, there were individuals from endemic areas, and presumably infected with LF, if it is accepted that LF was not present in mainland Australia prior to the 1800s, living and working in an environment that had high populations of mosquitoes capable of transmitting LF—an environment eminently conducive to the transmission of LF. 

There was some debate as to which of these groups of immigrants were responsible for bringing LF into Australia. Between 1853–1862, around 50 Chinese men were hospitalised with what was termed leprosy; however, Joseph Bancroft was of the opinion that some of these patients may have been misdiagnosed or also carried LF [7,53]. Salter, however, argued that the distribution of LF in tropical areas of Australia indicated that Pacific Islanders, restricted to these areas, were the source of transmission [7]. It is generally accepted now that LF was initially introduced from China in the 1850s, and again in 1861 when Pacific Island indentured labourers were introduced, and reintroduced from both locations over the next few decades [7,52].

In south-east Queensland, Brisbane appeared to be a focus of infection, with 40 cases recorded between 1891–1893, and a further 60 from 1898–1903. There was a noted seasonal variation, with a higher number of mf observed in the blood during summer. This is likely due to seasonal variation in mosquito populations, with higher rainfall in summer providing more breeding sites for mosquitoes. The higher numbers of mf in summer are therefore due to the repeated biting of mosquitoes due to the increased population of mosquitoes in the summer months [7]. A survey that was performed on 600 patients admitted to the Brisbane General hospital in 1904 identified a 15% prevalence of mf [7,8]. Subsequent surveys at the hospital in 1908 identified a prevalence of 10.8% (*n* = 1200), 11.5% in 1910, and 5% in 1911 [7,8]. Between 1922–1924, a more comprehensive survey, incorporating patients from northern Queensland as well as Brisbane, was performed. The highest number of examined individuals were from Brisbane, where the highest prevalence of mf of 3.6% was recorded, followed by Rockhampton, a city in central Queensland, where a prevalence of 3.4% was reported [54]. Certainly, LF was a major problem in Queensland and northern NSW at this time. Interestingly, one of the highest prevalences in the Brisbane area was from Stradbroke Island Aborigines with 4.4% infected (*n* = 45), and Purga Aboriginal Mission with 5.1% infected (*n* = 39) [54]. The high prevalence in these indigenous communities was more likely due to the presence of high populations of mosquitoes resulting from the high number of mosquito breeding sites in these areas, rather than evidence of LF present in Aboriginal individuals prior to colonisation. 

The prevalence of LF began decreasing in surveys from the late 1930s onwards, with a 0% (*n* = 228) prevalence of mf in patients from Brisbane General Hospital in 1938, although in 1944, a prevalence of 6.7% (*n* = 252) was recorded in the mental health hospital, Goodna. However, by 1949, the prevalence had fallen to 3.9%. Between 1937 and 1956, there were 56 admissions for LF, but all were negative for mf, indicating an old infection with no reproduction occurring [8]. The last active cases identified were in 1937 with two Australian Aboriginals being mf-positive sometime between 1949 and 1956 from islands in the Torres Strait, and a Mackay man in 1956 who had high numbers of mf [7,8]. 

In addition to the islands of the Torres Strait, Australia also has two other island territories. These are the Territory of Cocos (Keeling) (population 544), and Christmas Island (population 1843), both in the Indian Ocean. Christmas Island is closer to Indonesia (350 km) than Australia (1550 km). LF has not been recorded in either island group, but the closeness of Christmas Island to Indonesia, which is endemic for LF, may provide a potential source of infection. *Culex* mosquitoes capable of transmitting LF are present on the island.

### 6.2. Discovery of the Adult Parasites

Closer to south-east Queensland, where Dr. Joseph Bancroft was practicing medicine, the first reports of mf (the only known life-cycle stage at this time) in the area came from Dr. John Mullen, who described a case of chyluria (a rare condition in which lymphatic fluid leaks into the kidneys and turns the urine milky white) in Fortitude Valley, Brisbane, and by Dr. Thomas Rowlands, who in 1874 identified mf in the urine of a patient in Ipswich, a city to the west of Brisbane [7,55]. In 1874, Bancroft began isolating the mf from the blood of patients, which he subsequently preserved and sent to Dr. Roberts in Manchester and later, when the initial samples were destroyed in the post, to T. S. Cobbold, who identified the mf as well as an egg capsule, hypothesising that the adult worms must live in the human host [56,57]. Subsequently, Bancroft isolated female adult worms from a lymphatic abscess, and later from the hydrocele of a patient (1877), sending them to T. S. Cobbold, who published the finding in 1877 [56,57,58], naming them *Filaria bancrofti*. Dr. Bancroft’s discovery of the adult worms only preceded the same discovery by Lewis in India by seven months, and by de Silva Araujo in Brazil by nine months [7]. Discovery of a complete male adult specimen did not occur until 1888, although fragments had been found as early as 1879 [55,59]. 

### 6.3. Discovering the Vector

Bancroft wondered how such parasites living in the blood might be transmitted and hypothesised the involvement of a mosquito vector, a hypothesis also put forward by Manson [50]. To explore this idea further, he took an infected patient to his home, ‘Kelvin Grove’, named after the gardens of the same name in Glasgow, and later used as the name of the suburb of Brisbane in which Bancroft lived. Bancroft allowed *Aedes vigilax* mosquitoes to feed on the patient. It is not stated explicitly that mf were recovered from the mosquitoes by Bancroft, but in a letter to Cobbold, Manson was able to find mf in the stomach of mosquitoes in his own experiments. Despite the use of *A. vigilax* in Bancroft’sexperiment, *C. quinquefasciatus* (*Culex fatigans*) appears to have been the main transmitter of LF historically in Australia, although *A. aegypti* was also present in Brisbane at this time [7,60,61]. Initially, it was believed that the mosquito transported mf picked up from the blood of an infected individual to water, where the mf presumably matured and were ingested with water, thus infecting the human host. Elucidation of the role of the mosquito as a vector transmitting the mf to humans, rather than merely as a means to transport the mf to water, was only fully completed by Joseph’s son, Thomas Bancroft, in 1901.

Elucidation of the transmission of LF by a mosquito took a number of years of research by both Sir Patrick Manson and Dr. Thomas Bancroft. Thomas Bancroft was initially skeptical about the mosquito as a vector, but nonetheless set out to reproduce Manson’s work. Some of the issues regarding experimentation on mosquitoes as hosts were due to misconceptions, including that these insects only fed once [62]. As Thomas states, ‘It never occurred to us that our mosquitoes wanted to be fed, consequently they died of starvation about the sixth day, and before the filariae had developed sufficiently’ [7,62,63]. While residing in Burpengary, to the north of Brisbane, Thomas Bancroft corresponded with Manson regarding development of the filarial worms and continued research on the mosquito as a vector. To this end, using a grant from the British Medical Association, he employed a young servant, infected with LF, to be bitten by mosquitoes and found that it took 16–17 days for the larvae to mature. At this stage (1899), it was still considered by many, including Thomas Bancroft and Manson, that the mosquitoes merely introduced the filariae to water, and that LF was transmitted by drinking contaminated water [52,62,64]. Shortly after showing that the filariae died after only a few hours in water, Thomas Bancroft explored the idea that infection was due to swallowing an infected mosquito, before deciding that the filariae might gain access to the human blood stream during the act of feeding: ‘It has occurred to me that the young filariae may gain entrance to the human host whilst mosquitoes bearing them are in the act of biting. The entrance of warm blood into the mosquito may excite the young larvae, causing them pass down the proboscis into the human skin’ [7,62,63]. In 1901, Thomas Bancroft then demonstrated the presence of filariae in the proboscis of the mosquito [61], thereby implicating mosquitoes in the direct transmission of filariae to humans through feeding. 

## 7. Epidemiology

Both Joseph and Thomas Bancroft were highly vocal in advocating control measures for LF. Joseph Bancroft was primarily concerned with water safety and preventing mosquitoes laying eggs in water by closing rainwater tanks, or boiling or filtering water, while Thomas Bancroft promoted the use of mosquito nets, particularly for infected individuals [61,65]. A number of mosquito control measures were eventually undertaken, including the destruction or screening of containers, improved drainage, the destruction of breeding areas, and fish stocking. These control measures were undertaken in 1911 by the Brisbane City Council (BCC), which had, at the time, only been recently amalgamated from several smaller authorities [60]. The BCC was given responsibility for mosquito control by the State of Queensland. Between 1900 and 1910, active transmission occurred near the General Hospital in Brisbane (now the Royal Brisbane and Women’s Hospital in the suburb of Herston), where there was both a concentration of infected patients and breeding sites for *C. quinquefasciatus*. Once mosquito control measures began to impact the number of mosquitoes present, transmission was reduced and, eventually, LF was eliminated [7]. 

LF continues, however, to be a persistent public health problem outside of Australia, and research into LF has continued in Australian research institutes and universities including, as earlier indicated, at James Cook University and the laboratory of the late Dr. Rick Speare [47,66,67,68,69,70,71]. The most recent LF cases identified in Australia have all been in immigrants, refugees, and returned travelers coming from endemic areas [9], while the last recorded case of locally-acquired LF was in Mackay in 1956 [7].

## 8. Current LF Prevalence in Oceania 

In 1997, the highest recorded prevalence of LF worldwide (29.11%, 1.80 million) was in the Pacific Islands, including the Cook Islands, Fiji, French Polynesia, Guam, Kiribati, Marshall Islands, Micronesia, Nauru, New Caledonia, Niue, Palau Islands, PNG, Solomon Islands, Tonga, Tuvalu, Vanuatu, and Western Samoa [72] (Figure 2), although the highest number of infections occur in Asia (62.35 million) and Africa (50.57 million). Many of the Pacific nations are part of PacELF, which has been highly successful, and many of the Pacific countries previously endemic for LF are now well on the way to the goal of elimination by 2020, with current prevalences well below 1%.

### 8.1. Active Transmission

#### 8.1.1. Papua New Guinea (PNG)

PNG is the closest neighbouring country to Australia (Figure 2); historically, it was endemic for LF prior to the British colonisation of Australia, and continues to be endemic. As already mentioned, James Cook University has been involved over the past two decades in the elimination of LF and is designated as a WHO collaborating center for the control of LF, recently expanded to include soil-transmitted helminths (STH) and other neglected tropical diseases [73].

There are limited available reports of the current LF prevalence levels in PNG. A research study in 14 villages in Dreikikir district, utilising diethylcarbamazine and/or ivermectin, as part of an MDA, reduced the prevalence from 47% (*n* = 797) in 1994 to 1% (*n* = 750) in 1998, showing that MDA might be sufficient for causing transmission interruption in areas with low to moderate endemicity [74]. A more recent study investigating the mosquito vectors, specifically *Anopheles* species, used volunteers from an endemic village to determine the level of uptake of mf by mosquitoes [75]. Individuals from the village were screened and LF antigen-positive individuals were requested to provide a blood sample, which was subsequently examined for mf and then used to feed mosquitoes in the laboratory [75]. The mosquitoes were dissected and examined for mf [75]. All three *Anopheles* species utilised in the study proved efficient in the uptake of mf [75]. A comprehensive literature review showed LF prevalences of 30.4–64.7% for the period 1983–1992, 30.1–56.9% for the period 1993–2000, and 7.8–12.8% for the period 2003–2011, indicating a downward trend in the most recent time period [11]. The same study estimated the at-risk population resident in the endemic areas to be 4.81 million (70.4%). 

Research programs in PNG have also included examining the use of insecticide-treated bednets in order to help prevent transmission [40]. A study that investigated the effect of bednets in reducing the incidence of LF infection in three PNG villages found a large reduction in mf prevalence in mosquitoes after bednets were introduced. In 1998, after five years of MDA, the prevalence was reduced to 3.7–10.8%. Prior to the introduction of bednets, the human prevalence was 23.7–38.6%, emphasising that ongoing mosquito control is as essential for elimination as chemotherapy [40]. Mf prevalence in *Anopheles punctulatus* mosquitoes decreased from 1.8 to 0.4% after the bednets were distributed; however, MDA was also occurring in the villages, which would also account for the lower mf uptake by mosquitoes as there should have been less live mf in treated individuals [40]. The benefit of the nets was primarily reflected in a decrease in reported bites from 6.4–61.3 to 1.1–9.4 bites per day.

It is clear that the prevalence of LF has been considerably reduced from initially very high levels in PNG, likely as a result of MDA and mosquito control [76,77,78]. PNG joined the PacELF program in 2005. Initial baseline prevalence in 2006 in six provinces ranged from 28.36% in Bougainville to 32.71% in East New Britain. There was one province, Oro, where prevalence at the baseline was significantly lower than the other provinces, at 1.26%. Since then, MDA has been carried out initially in all six provinces, before being reduced to five provinces in 2010–2013 [79]. In concert with MDA, diethylcarbamazine-medicated salt was also introduced in some areas [19,79]. Previously medicated salt had been used successfully in China, India, and Tanzania for LF control. In PNG, however, it faced some problems as most salt for cooking was acquired from cooking food in sea water and local health workers had been trying to reduce salt intake for heart health [19]. 

#### 8.1.2. The Indonesian Province of Papua (Originally Irian Jaya)

Western New Guinea (WNG), also known as Papua (formerly Indonesian Irian Jaya) and West Papua, is located at the western end of the island of New Guinea, with the eastern end of the island being PNG (Figure 2). WNG was annexed by Indonesia in 1962 and is the only Indonesian territory to be situated in Oceania. It has been included in this review, despite being nominally part of South East Asia rather than the Pacific group, due to its closeness to Australia and PNG, sharing of a land border with the latter, and its similarity in terms of the environment. Historically, *W. bancrofti* was highly prevalent in WNG, particularly in prisoners in FakFak during the Second World War—likely due to the concentration of infected persons and the presence of susceptible mosquitoes [8]. As of 2012, an estimated 113.2 million individuals required treatment for LF in Indonesia [80].

The three species that cause human LF (*B. malayi*, *B. timori*, *W. bancrofti*) occur in Indonesia, and LF cases occur in all provinces; WNG has some of the highest rates of LF in Indonesia [81]. There are few contemporary manuscripts detailing prevalence in West Papua, although filariasis is still highly prevalent elsewhere in Indonesia [82]. In 2009, Papua had the third highest incidence of LF with 1158 recorded cases [83], while in 2015, West Papua had 1244 cases, followed by Papua with 1184 [84]. Clinical cases actually increased in Indonesia between 2000 and 2009, although this was likely due to increased awareness and active searching for the disease, particularly after 2002, when the national LF program commenced [83,85]. Between 2010 and 2015, the number of clinical cases remained fairly consistent (11,969–13,032), although there was an overall increase in infected individuals, including a high peak of nearly 15,000 cases, indicating that it is still of high public health importance there [84]. Indonesia is part of the global initiative to eliminate LF by 2020 and undertakes annual MDAs (of five to six rounds yearly) and mosquito control in an effort to achieve this goal [85]. There have been some implementation and compliance issues concerning the MDAs, with an average coverage of 39.4% in 2010, increasing to 73.9% in 2014, before decreasing slightly to 69.5% in 2015 [81,84]. Low compliance is due to a number of reasons, including asymptomatic infections with individuals considering treatment to be unnecessary, the fear of side effects (including among pregnant women), taking too many drugs, the lack of trust towards those distributing the drugs, infrastructure problems, the reliability of databases, and the poor training and competence of health workers [81,85,86]. 

#### 8.1.3. Timor-Leste (East Timor)

Timor-Leste is a small island nation in South East Asia to the north of Australia and makes up half of the island of Timor (Figure 2). It has been included in this review due to its closeness to Australia, and also due to the presence of Australian peacekeepers during 1999–2002. This was in accordance with United Nations resolutions to assure safety and civil order after a referendum was conducted for the East Timorese to vote for independence from Indonesia, which annexed the country in 1975, prior to which Timor was under the control of Portugal. The vote was ultimately overwhelming in favour of independence, after which violent clashes occurred, stirred up by pro-Indonesian militia. At this stage, a UN peacekeeping force was introduced and this was largely made up of Australian soldiers. 

Two agents of LF, *Brugia timori* and *W. bancrofti*, occur in Timor-Leste, both of which are nocturnally periodic. The earliest study on LF in Timor-Leste occurred in 1958 and recorded a prevalence of 2% (*n* = 3350) for a species described then as *B. malayi*—later classified as *B. timori*. Blood was also collected at night from 48 individuals, finding a prevalence of 10.4% [8]. 

In the mid 1960s, the two forms of filariasis in Timor were identified and separated. It was at this time that the *Brugia* species in Timor was acknowledged to be different morphologically to the usual *B. malayi* and was called the ‘Timor mf’. At the same time, the presence of *W. bancrofti* was also confirmed. 

In 1964, a survey recorded a prevalence of 7.4% for *B. malayi* (*timori*) only infections, 2.6% *W. bancrofti* only, and 1.7% were infected with both species [8]. Later studies in the 70s, and more recently in 2002, confirmed that while *W. bancrofti* accounted for 90% of LF cases worldwide, in Timor-Leste, the dominant species is actually *B. timori*. In 2007, the prevalence of LF was 2.6% (*n* = 3461), although the prevalence may have been underreported due to the low sensitivity of the diagnostic procedure used, involving antibody detection in urine.

A more recent national survey (2011–2012) conducted by the Timor-Leste government in conjunction with AusAID and WHO, examined blood samples taken from fingerpricks for the prevalence of mf, and recorded a prevalence of 17.5% for *B. timori*. This survey covered 13 districts, collecting 2164 blood samples [87]. Prevalence was high in all villages, with the lowest recorded prevalence being 10.3%, and a number of children <5 years of age were seropositive, indicating that high transmission rates were still occurring. Indeed, prevalence was higher in nearly all villages in 2012, compared with the national survey results in 2002. This may have been due to an increased number of samples collected in 2012 and actual bias in the sample collection [87].

Serology undertaken on Australian soldiers involved in the peacekeeping mission from 1999–2002 indicated that they were certainly exposed to filarial nematodes, although all were asymptomatic, and it was unclear if any had progressed to an established or patent infection [88]. 

#### 8.1.4. Samoa (Formerly Western Samoa) 

Samoa is comprised of two islands to the north-east of Australia (Figure 2), with a third island, American Samoa, under the control of the USA, lying close by. The earliest reports of elephantiasis in Samoa were made in 1878, noting the ‘frequent occurrence’ of patients with lymphoedema in the legs and genitals [8]. In 1923, the earliest recorded prevalence survey for LF identified an mf prevalence of 28.7% (*n* = 4294) and an elephantiasis prevalence of 2.7%. By 1945, these prevalences had not changed significantly (mf 19.2–24.1%, 3.6% elephantiasis). The first LF control program was instigated in 1965 as part of a pilot study by the WHO and UNICEF and comprised DEC chemotherapy weekly for six weeks, followed by monthly treatments for 12 months [8]. The coverage for this was 21% of the population and reduced the mf rate from 19.06% pre-treatment to 1.63% post-treatment. A second round of chemotherapy, comprising monthly doses for 12 months in 1973, further reduced the prevalence to 0.11%; there was a slight increase in the mf prevalence from completion of the first control program to 2.26% [8].

Samoa has been part of PacELF since 2001, when the country prevalence was 2.62%, although two areas had prevalences between 3.05–7.35% determined using ICT [79]. By 2006, the prevalence had been reduced to 0.36%. Despite the low prevalence, active transmission may still be occurring, as evidenced by a case of LF identified in 2011 in Australia in a Samoan man who had recently visited Samoa and presented with swelling in the right leg and scrotum [9]. However, it can take many years for overt symptoms to occur so it is unclear if he may have been infected from an earlier visit or if he had lived in Samoa as a child.

American Samoa joined PacELF in 1999; the baseline prevalence of 16.50% was reduced to 2.3% by 2007 after seven rounds of MDA [79]. A final round of MDA commenced in 2009, with active surveillance after this [79]. A study utilising serum samples collected in 2010 found a prevalence of 0.75–3.2% (>128 units, >32 units respectively) by the Og4C3 antigen ELISA, and 8.1% by Wb123 antibody ELISA [89]. Hotspots of transmission were identified in that study and a follow-up in 2014 confirmed that active transmission was still occurring [90].

### 8.2. Active Surveillance

#### 8.2.1. New Caledonia

The French territory of New Caledonia is a collection of islands that lie 1201 km east of Australia in the Pacific Ocean (Figure 2). LF has been endemic there since at least the 18th century, when the prevalence was as high as 59% in some villages [8]. In a survey of 382 individuals in 1999, 33.5% were seropositive for LF and 3.7% had mf present on blood smear examination. Two patients had clinical manifestations suggestive of LF, but neither had mf and only one was seropositive [91]. A more recent cross sectional survey in 2013 tested 1035 individuals and found a seroprevalence of 0.62% [92]. As two of the seropositive patients had never travelled outside of New Caledonia, it was considered that low-level transmission might be occurring. However, no mf were found in either patient and PCR was negative, which may indicate an earlier or past infection, or an immature infection [92]. Certainly, follow-up would be recommended.

#### 8.2.2. Tuvalu (Formerly Ellice Islands)

Tuvalu is a group of islands halfway between Australia and Hawaii in the Pacific Ocean (Figure 2). In 1923, when the islands were known as the Ellice Islands, an mf prevalence of 46% was reported in 1169 volunteers; in 1945, a smaller survey of 258 individuals found a prevalence of 34.1% [8]. 

Tuvalu joined the PacELF program in 1999. At the onset of MDA, implemented from 2001–2005, the baseline prevalence was 22.30%; this was reduced to 3.4% in 2007–2008 [79].

#### 8.2.3. Micronesia

Micronesia is a collection of thousands of islands to the north of Australia (Figure 2). Micronesia comprises five sovereign nations, the Federated States of Micronesia (FSM), Palau, Kiribati, Marshall Islands, and Nauru, as well as three United States of America territories—Northern Mariana Islands, Guam, and Wake Island. Seemingly, LF has historically been of low prevalence or non-endemic on these islands [8]. Of the U.S.A. territories, Guam has had no reported incidence, whereas in the Mariana Islands, 13.5% (*n* = 243) of individuals had mf on blood smears; however, the parasite was only found on one of the islands and has been considered non-endemic since the start of the PacELF [79]. Only two islands in the Marshall Island group have historical LF data, with mf prevalence in individuals being 1% and 3.6%. Palau had a reported mf rate of 12.6% in 1967, which was reduced to 0.3% after MDA in 1970 [8]. Nauru, now considered to be non-endemic for LF, had a historical prevalence of 36.1% in 1933 [8].

The FSM were thought to be non-endemic for LF after MDA in the 1970’s. In 2002, a survey of 50 children in a remote atoll of FSM identified 38% as being seropositive. A follow-up survey in the following year also examined adults (*n* = 253), finding 38% seropositivity in the whole community and an mf prevalence of 22% [93]. Transmission was obviously still active at this site, reinforcing the need to continue surveillance in at risk areas. At the time, the local inhabitants reported an increase in the mosquito population, which may have accounted for the increase in LF transmission—although LF still had to be present in the community for transmission to occur; as a result, the local residents embarked on mosquito control themselves [93]. 

The following nations and territories in Micronesia are still implementing or require targeted treatment according to the Pacific Technical Report by the WHO [79]: Palau, FMR, Marshall Islands, and Kiribati. All other nations and territories are considered non-endemic for LF.

#### 8.2.4. Fiji

Fiji lies to the east of Australia and comprises more than 300 islands (Figure 2). Historical reports of LF in Fiji began in 1876 when elephantiasis was common amongst Fijians, particularly those who lived in marshy areas [8]. In 1905, the prevalence of mf in the blood of individuals was recorded as 25.7% (*n* = 608), and in 1912, there was a prevalence of 27.1% (*n* = 1320), indicating high-level transmission [8]. This continued through to 1944, when the largest survey undertaken to that point identified a prevalence of 14.2% (*n* = 57,888). Vector control began in 1961, with a pilot study finding a reduction in mf prevalence from 12.1% to 2.7% after its commencement in the area [8].

Fiji has participated in ongoing MDA since joining the PacELF in 2001. Baseline prevalence for PacELF by antigen detection in 2001 was 15.17%, reducing to 9.50% in 2007 post-MDA which involved five yearly rounds of chemotherapy. The mf prevalence in 2007 was 1.40% [79]. The uptake of chemotherapy has been very successful in Fiji, with coverage in 2012 being 94% [79,94].

### 8.3. Elimination Achieved

#### 8.3.1. Solomon Islands 

The Solomon Islands lie to the north-east of Australia (Figure 2) and have a history of LF, although the disease is considered eliminated today—possibly as an added benefit of mosquito control for malaria eradication. A single case of elephantiasis, reported in 2011, was thought to have been acquired some years earlier and not as a result of active transmission [71]. Surveillance of villages near where the patient lived found only one other person positive for LF, but no mf were detected [71]. 

The highest prevalence of LF in the Solomon Islands was recorded in 1945 and ranged from 10.2–31.5% by village, which was similar to the 28.5% prevalence recorded in 1965 [8]. During the 1960s and 1970s, there were several attempts made at controlling the malaria vector, *Anopheles farauti*, which would have impacted LF transmission as it is also a vector of LF. Treated bednets were introduced in 1992 and more recent measures have included indoor residual spraying. While malaria is still endemic in the Solomon Islands, vector control for malaria has seemingly resulted in the elimination of LF.

#### 8.3.2. Republic of Vanuatu 

Vanuatu is an Island group comprising around 80 islands that lie to the north of New Caledonia and 1750 km east of Australia (Figure 2). Vanuatu was originally known as the New Hebrides, so named by Captain James Cook, and was managed by the French and British before becoming an independent country in 1980. 

MDA was implemented in 2000 by the Ministry of Health with the aim of eliminating LF as a public health problem by 2004, when the MDA was targeted to cease if prevalence was <1% [95,96]. Historical reports of LF in Vanuatu are scarce and likely grouped together with reports from the New Hebrides. In 1927, the reported prevalence was 31% (*n* = 318) [95,96]. A survey was performed in 1998, two years prior to the commencement of MDA, and again in 2002, two years after the commencement of MDA as part of PacELF. The follow-up survey in the 2002 study focused on the four provinces with the highest prevalence at baseline and examined blood from 572 individuals. Seroprevalence in 1998 was 22% compared to 8% in 2002, and mf prevalence was 11% in 1998 and 0.8% in 2002 [95]. These dropped further in a 2005/6 survey to a seropositivity of 0.16% and 0% mf prevalence [96]. The most recent survey in 2012 reported only two cases. Surveillance is ongoing to ensure that the transmission of LF is no longer occurring [97]. Vanuatu is therefore well on the way to declaring the elimination of LF. In addition to MDA, bednets are utilised to prevent not only LF, but also malaria, which is prevalent. An additional component is health education, which appeared to ameliorate many of the concerns causing poor MDA compliance in other pacific countries [95]; MDA compliance ranged from 75.5–81.5% over the five years of the MDA (2000–2004) [96]. As of 2016, LF is considered to have been eliminated from Vanuatu [98].

#### 8.3.3. Tonga 

Tonga lies to the east of Australia and is made up of 170 islands in the Pacific Ocean (Figure 2). The first report of LF in Tonga was that by Captain James Cook in 1785, who noted the occurrence of elephantiasis (described as the swelling of legs, arms, and scrotum). In 1896, the prevalence ranged from 20–46.9%, depending on the location, and in 1965, ranged from 28.2 to 49.6% [8]. At the beginning of the PacELF program in 2000, the prevalence was 2.7%, which was reduced to 0.38% in 2006. Elimination of LF from Tonga was declared in 2017 [98].

#### 8.3.4. Cook Islands 

The Cook Islands comprise 15 islands in the South Pacific (Figure 2) and have historically had a very high prevalence of LF. In 1925–1926, the mf prevalence ranged from 26% and 54.8% across the islands. High prevalence continued through to 1949 when the recorded prevalence was still as high as 42.5%. MDA commenced in 1969 on Aitutaki and was expanded thereafter, reducing the prevalence to 0.2% in 1971 [8]. In 1999, the Cook Islands joined PacELF; the prevalence then of LF by ICT as part of PacELF was 8.60% and in 2007, the prevalence by ICT was 0.33% after yearly multiple rounds of MDA, with only two islands returning positive results. These islands were targeted with further MDA. Elimination was declared in 2016, although active surveillance continues [79,98]. 

#### 8.3.5. Niue 

Niue (current population 1624) is a small self-governing state in association with New Zealand to the east of Tonga and south of American Samoa (Figure 2). In 1954, there was an mf prevalence of 22.1% (*n* = 748), after which MDA commenced and in 1956, the prevalence had beenreduced to 2.9% (*n* = 2791) [8]. In 1972, a second MDA program was implemented, prior to which the prevalence was 16.3% [8]. Niue joined the PacELF program in 1999, when the prevalence was reported as being 3.12%. Surveys after four years of MDA recorded a prevalence of 0.23%. As of 2016, LF was considered eliminated from Niue [98].

## 9. Immigration to Australia

In 2016, the top ten countries from which individuals emigrated to Australia were, in order of highest to lowest number, the UK, New Zealand, China, India, the Philippines, Vietnam, Italy, South Africa, Malaysia, and Germany [99]. Of these, India, the Philippines, and Vietnam are endemic for LF, while Malaysia and China have eliminated the disease. South-East Asia, which includes the Philippines and Vietnam, as well as other countries with relatively high rates of migration to Australia, such as Cambodia and Myanmar, account for roughly 15 million cases of LF [80]. As of 2012, 41.7 million individuals required treatment in Myanmar for LF, 29.4 million in the Philippines, and 73,495 in Thailand, while Vietnam is under surveillance and approaching elimination [80]. Health screening of immigrants and refugees into Australia does not include testing for LF. Thus, it is likely that cases without obvious symptoms or physical manifestations will not be identified upon entry to Australia. As LF is not a notifiable disease, it is unclear how many cases of LF have been brought into Australia by immigrants, refugees, and returned travelers. Relatively recent cases of LF diagnosed in Australia have been contracted in Myanmar, India, and Samoa [9].

## 10. Mosquito Hosts for LF

### 10.1. Host Species and Distribution in Australia (Including Islands of the Torres Strait)

There are a number of mosquito genera capable of transmitting LF, including species from the genera *Culex*, *Anopheles*, *Mansonia*, and *Aedes*. *Aedes aegypti* transmits dengue in North Queensland and can also transmit LF, although early reports of mosquito LF transmitting efficacy initially indicated that it was not involved [8,100]. Thomas Bancroft first identified *Ae. aegypti* as a vector in the transmission of dengue in 1906 in Brisbane. Dengue was only eradicated from Brisbane in 1948 [60]. Dengue is now not endemic in Australia and outbreaks are generally due to an infected person (or infected mosquito) entering the country. In 2017 [101], there were three outbreaks of dengue in North Queensland, with a total of nine confirmed cases. There have been larger outbreaks since 2000, with nearly 900 cases recorded between 2003 and 2004, and 890 cases between 2008–2009 [102]. These outbreaks indicate that this is a still a large problem despite ongoing mosquito spraying and community education towards reducing the habitats of *Ae. aegypti*. The *Ae. aegypti* mosquito breeds in water containers close to houses, including pot bases, birdbaths, and puddles of water in tarpaulins, which help bring the mosquito into contact with humans to transmit diseases [103]. This mosquito species was largely removed from Brisbane in the early 1900s due to advances in water supply, namely, the introduction of a large water reservoir that reduced the number of water tanks, and council ordinances that rainwater tanks needed to be covered with all openings covered in mesh, therefore reducing breeding sites for mosquitoes [7,60]. 

There are a number of *Aedes*, *Anopheles*, and *Culex* species present in Australia, and a single species of *Mansonia*. The vector status of many of these species is unknown, but many bite humans only rarely and are therefore unlikely to be vectors; other species primarily inhabit swamps and bushland, which would reduce their effectiveness in transmitting LF or other vector-borne diseases. 

*C. quinquefasciatus* is capable of transmitting LF and is also present in Australia; unlike *Ae. aegypti*, which is now restricted to Queensland although previously found in NSW, *C. quinquefasciatus* is present in all states and territories of Australia [104]. Historically, this mosquito, which has a very broad range and is present worldwide, was the main transmitter of LF, although it may have been quite inefficient in this regard, which may have contributed to the eventual elimination of LF in Australia [7,8]. Its breeding habitats include ditches, drains, and septic tanks and it is common in urban areas with poor drainage and sanitation [105]. Other species capable of transmitting LF present in Australia are: *An. bancroftii* (which may also be capable of transmitting malaria), *An. farauti*, *An. amictus*, *Ae. kochi*, *Ae. vigilax*, *M. uniformis*, *C. annulirostris*, and *C. bitaeniorhynchus*. *Aedes albopictus*, an invasive mosquito species not native to Australia, has previously been introduced into the islands of the Torres Strait [106] and mainland Australia [107], and has also become established in Asia, Europe, Africa, and the Americas. *Aedes albopictus* is an important mosquito species due to its successful establishment in many parts of the world and its ability to act as a vector of both LF and dengue. This mosquito is no longer present in Australia due to successful control programs. However, it is still present in PNG and Indonesia, and thus colonisation could potentially occur in the future if mosquito control programs are halted.

### 10.2. Vector Competence—Are Some Species Better Than Others at Transmitting LF

Transmission of LF is quite inefficient, with upwards of several hundred to thousands of infective bites required for transmission to occur [108]. Different mosquito species also have varying efficiencies and in general, there is lower vector efficiency observed in the Pacific and Oceania than observed in Africa [108]. In early studies in Tanzania and Liberia, where the vectors were *C. quinquefasciatus* and *An. gambiae* in Tanzania and *An. gambiae* only in Liberia, upwards of 11,000 infective bites were required to induce microfilaraemia. The lowest number of bites was 269, also in Tanzania. In comparison, the highest required number of bites in Asia/the Pacific was 67,568 infective bites in Fiji (*Ae. polynesiensis*), followed closely by 57,803 in Indonesia (*C. quinquefasciatus*). The lowest number was 142 in Malaysia, where the infective species was *B. malayi* (*Mansonia* spp.) [108]. A factor that could greatly influence this is that the mf are not injected into the blood as is the case in malaria, but are introduced onto the skin, with the mf then making their way into the bite wound and thus into the blood [14]. The skin is a generally harsh environment for micro-organisms to survive in; it can be quite dry, and secretes substances which can cause the skin to become more acidic and also damage or cause death. Therefore, transmission is less straight forward than occurs with vector-borne pathogens that are injected directly into the blood.

Some mosquito species are more efficient at transmitting LF than others. Vector competency relies on the uptake of mf from the infected human host, development in the mosquito to the infective L3 larvae, and transmitting those infective larvae to humans [109,110,111,112,113]. Certain species of mosquito are able to transmit parasites from humans harbouring low levels of mf, whereas other species can only transmit when high numbers of mf are present; paradoxically, very high levels of mf have been associated with the early death of mosquitoes [109,114]. The mf can also demonstrate nocturnality [115]; thus, mosquitoes taking a blood meal during the day are likely to encounter low parasitaemia which, depending on the mosquito species and when they are active, may limit transmission. There is a strain of *W. bancrofti* in the South Pacific which does not appear to exhibit nocturnal periodicity and mf can be found at any time of the day [8]. Additionally, the time of day that the infected mosquito is feeding will affect when mf can be seen in the blood, with peak mf levels in the blood observed during the peak biting periods of the mosquitoes [116]. While some mosquitoes are classified as day biters, there is often peak biting in the early morning and evening, such as the case with *Ae. polynesiensis*. Some mosquito species do not feed on or bite humans, whereas other species that do bite humans will have varying degrees of ‘ferocity’, with some species giving more bites than others. *Ae. Polynesiensis* is considered a good vector of LF [117,118], likely due to a number of factors, including its high biting frequency, compared with other mosquitoes [118]. A high biting frequency will decrease the time required for infection to occur.

*Anopheles* spp. mosquitos cause injury to mf during uptake due to foregut apertures, which are less developed in *Culex* and *Aedes* spp. Mosquitoes, resulting in less damage to mf [111,112,114]. *Anopheles* spp. mosquitos also need to ingest high numbers of mf for gut penetration and subsequent development of the larvae to L3. However, results on mf uptake and damage vary between species, geographical location, and study design. A study on the vector competency of *Anopheles* species in PNG showed a considerable difference in ingested mf between *An. punctulatus* (4.2–23.7%), *An. farauti s. s.* (mf prevalence in *Anopheles punctulatus* 8.6%), and *An. hinesorum* (61.9–100%) at low and high density exposures, respectively [75]. The mf recovered from mosquitos were examined for damage during uptake, although *An. hinesorum* mf were only examined in low-intensity infections. As a proportion, more mf were damaged in low-intensity infections. *An. punctulatus* had the highest proportion of damaged mf in low-intensity infections, but the proportions of damaged mf were similar between *An. punctulatus* and *An. farauti* in medium and high intensity infections [75]. *An. farauti* exhibited low survivorship in direct correlation to the intensity of infection—in high intensity infections, survivorship of the mosquito was very low, with only 20% surviving to 14 days post-infection; survivorship was not examined in the other species [75]. The filarial vector competency of *Ae. aegypti* is linked to the age of the mosquito, with older mosquitoes being less efficient in transmitting filarial nematodes, although frequent blood meals can reverse this trend [119].

As indicated in older studies, it appears that *C. quinquefasciatus* is a poor vector for LF, which may have helped with the elimination of LF from Australia. However, it is possible that there are geographic differences between the mosquito species which may cause variation in LF-transmitting ability. One study compared *Ae. aegypti* and Haitian *C. quinquefasciatus* and found a much higher uptake of mf and development to L3 larvae in the former species, which had been considered a poor vector in older studies [100,104].

#### Climate and Potential Spread of Mosquito Vectors in Australia

Based on climate modelling (2000–2009), the eastern seaboard of Australia would provide suitable habitats for *Ae. albopictus*, while future predictions for climate change in Australia indicate a wider suitable range for both *Ae. albopictus* and *Ae. aegypti*, which were previously present in NSW, WA, and the NT [60,107,120]. In Europe, climate change has already impacted the transmission of vector-borne diseases by expanding tropical and subtropical zones and this has led to increases in the survivability zones for insect vectors, particularly mosquitoes. This has resulted in the spread of *Dirofilaria* species, zoonotic filarial nematodes which utilise mosquitoes as transmission vectors, into new areas in Europe [6]. Other species capable of transmitting LF are already present in Australia, such as *C. quinquefasciatus*, and *An. farauti*, which is a vector of LF in Papua New Guinea [11]. The introduction from outside Australia of *Ae. polynesiensis*, a competent LF vector, would be of concern. This mosquito is exclusively tropical, and climate change may increase its viable range in Australia. It is therefore important to prevent its spread, particularly as it can also act as a vector for dengue, chikungunya, and Ross River viruses [121,122,123].

## 11. Conclusions

LF appears not to have been endemic on mainland Australia prior to European colonisation, but was present in the islands of the Torres Strait, and was thought to be introduced several times by immigrants from China and the Pacific Islands. Relevant mosquito species that can transmit LF are present in all states of Australia. Immigration will continue to be a concern for the importation of new diseases including mosquito-transmitted infections such as dengue and chikungunya, as well as LF. Current control measures in North Queensland against *Ae. aegypti* for dengue, and in Brisbane against marsh mosquitoes, will likely benefit any future need for the control of LF as well. LF is a very poorly-transmitted disease, requiring the presence of a highly concentrated population of infected individuals for successful spread. The absence of a competent mosquito host such as *Ae. polynesiensis*, also reduces the risk for re-introduction to Australia. It is therefore very unlikely that LF will ever become re-established in Australia, with only sporadic reports of infections in returned travelers and, more likely, in immigrants and refugees from endemic areas.

The main area of risk to Australia for the re-introduction of LF remains the Torres Strait islands, which lie very close to endemic PNG in the north and mainland Australia in the south. There is already concern for the importation of other diseases such as TB from PNG via the Torres Strait [124]. However, MDA and mosquito control in PNG have also reduced the prevalence of LF there, thus decreasing the likelihood of re-introduction to the islands of the Torres Strait. 

The potential for the re-introduction of LF onto the Australian mainland seems remote. As LF caused by *W. bancrofti* can be readily treated with DEC, ivermectin, or albendazole, and coupled with the ongoing, highly successful mosquito control efforts against dengue and other mosquito-borne viruses, it seems very unlikely that LF would regain a foothold on the Australian mainland. Monitoring in the Torres Strait, however, should occur as the risk of infection introduced from PNG remains a threat, albeit at a low level.
